# Supplementary figures and images for: Efficacy and safety of three-dimensional magnetically assisted capsule endoscopy for upper gastrointestinal and small bowel examination
Source: PLoS One. 2024 May 7;19(5):e0295774. doi: 10.1371/journal.pone.0295774 (PMC11075891; doi:10.1371/journal.pone.0295774)

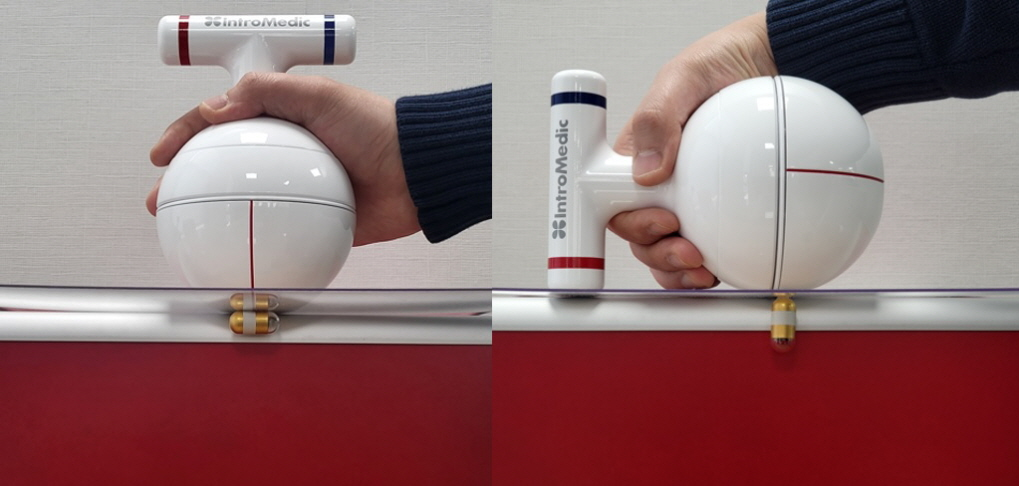

Supplement: S1 Fig — The camera direction of the 3D MACE could be adjusted using the direction of N and S poles on the handle of the controller. (TIF) [file pone.0295774.s001.tif]

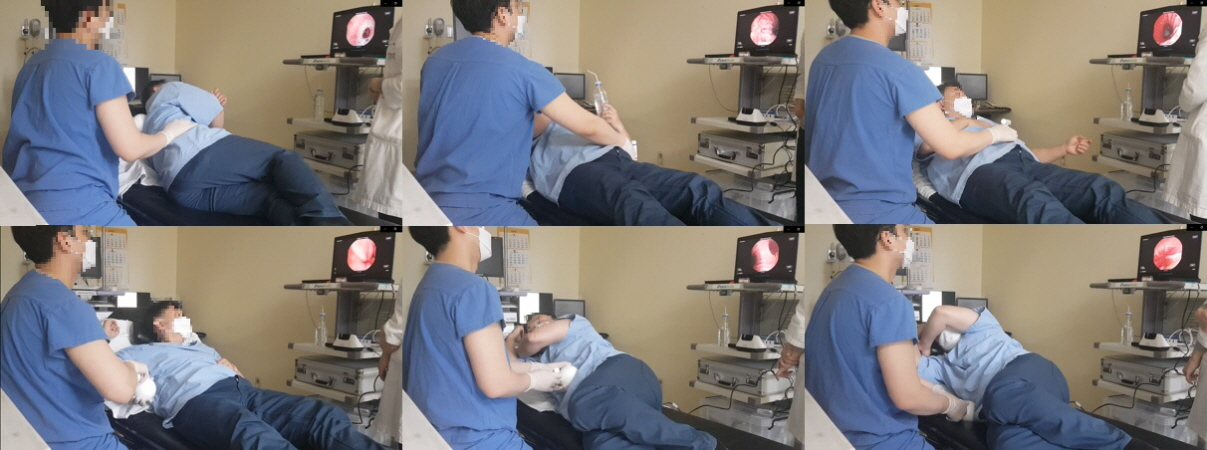

Supplement: S2 Fig — (TIF) [file pone.0295774.s002.tif]

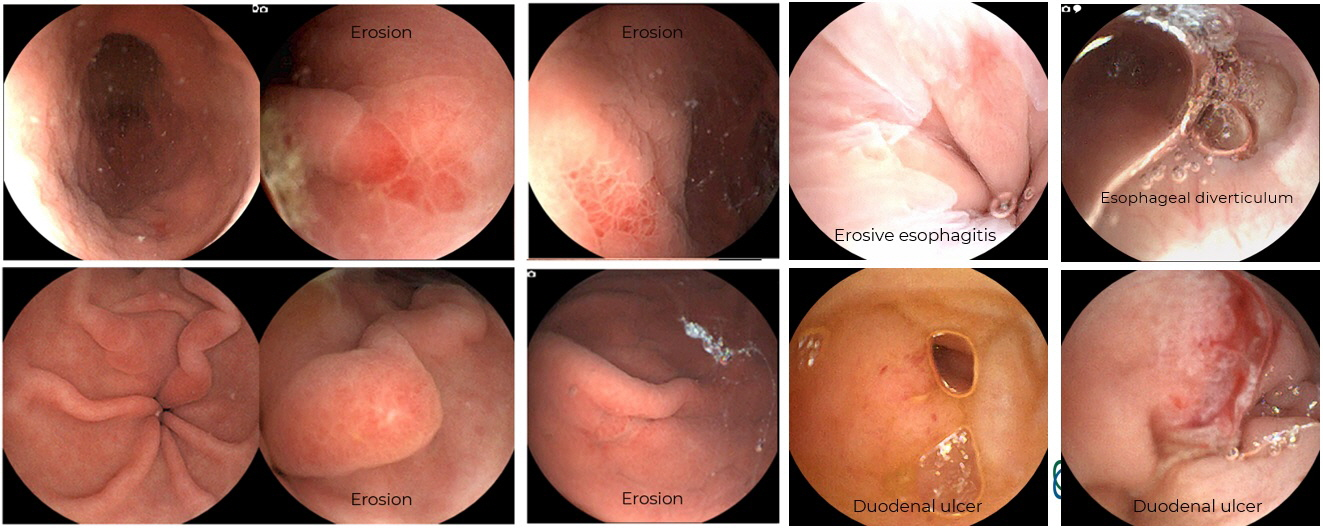

Supplement: S3 Fig — (TIF) [file pone.0295774.s003.tif]

# Lesion detection 3D reconstruction

Images of MACE and Upper endoscopy

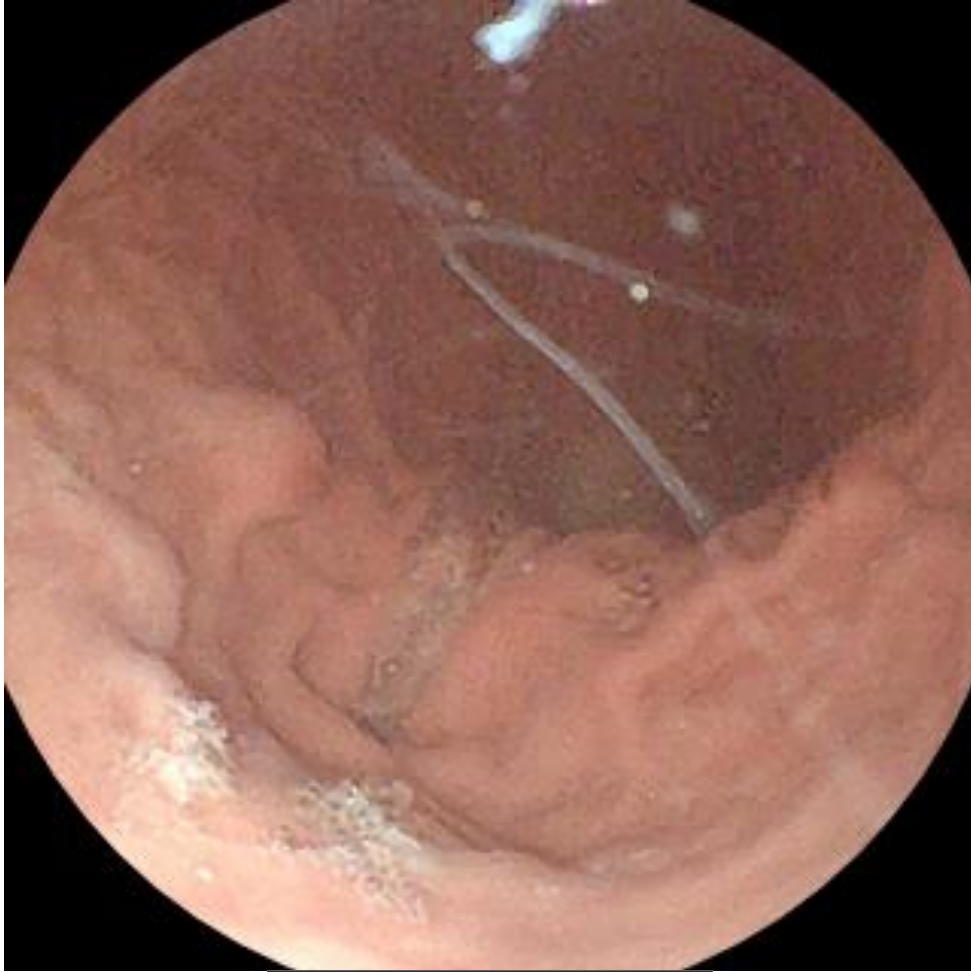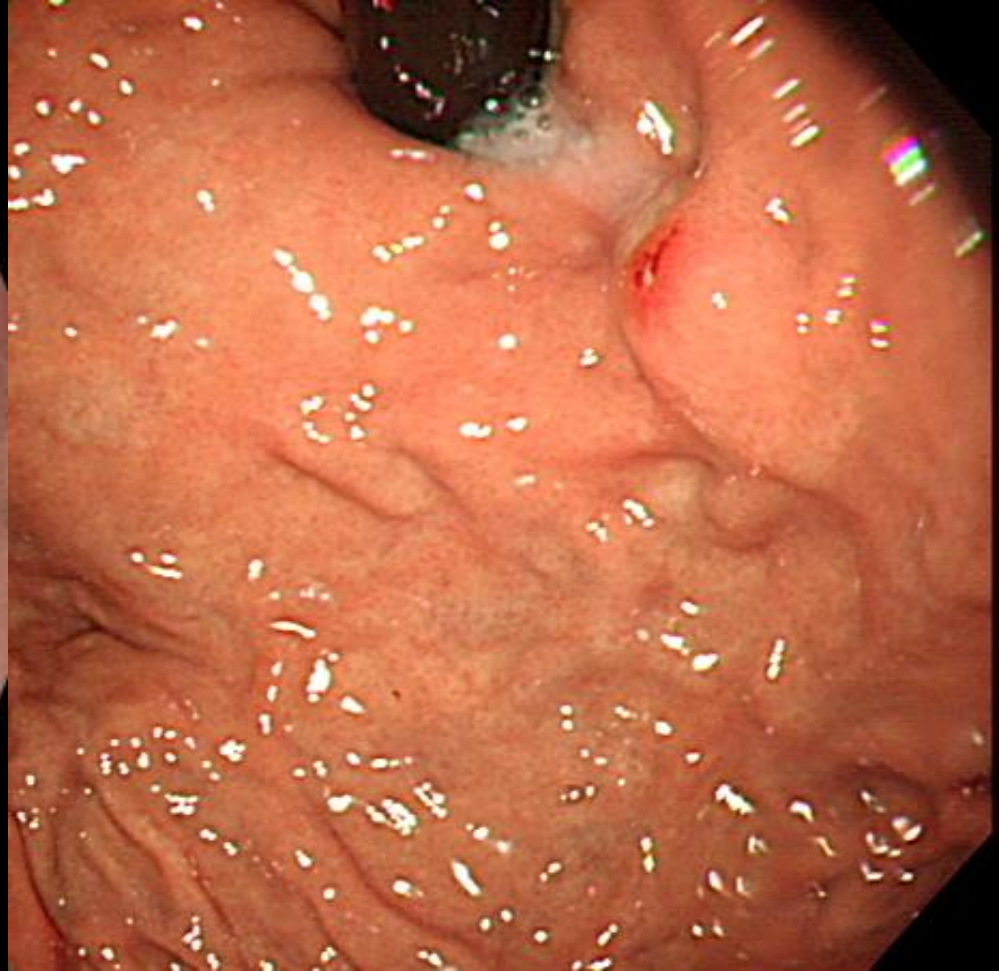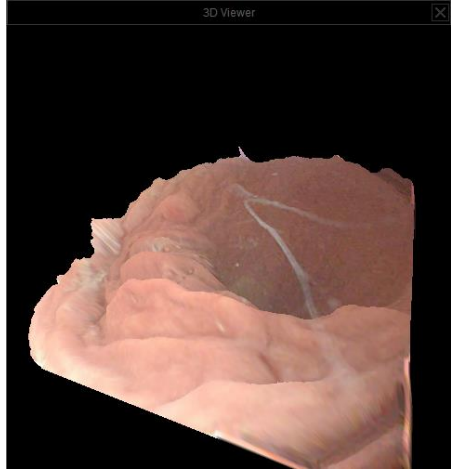

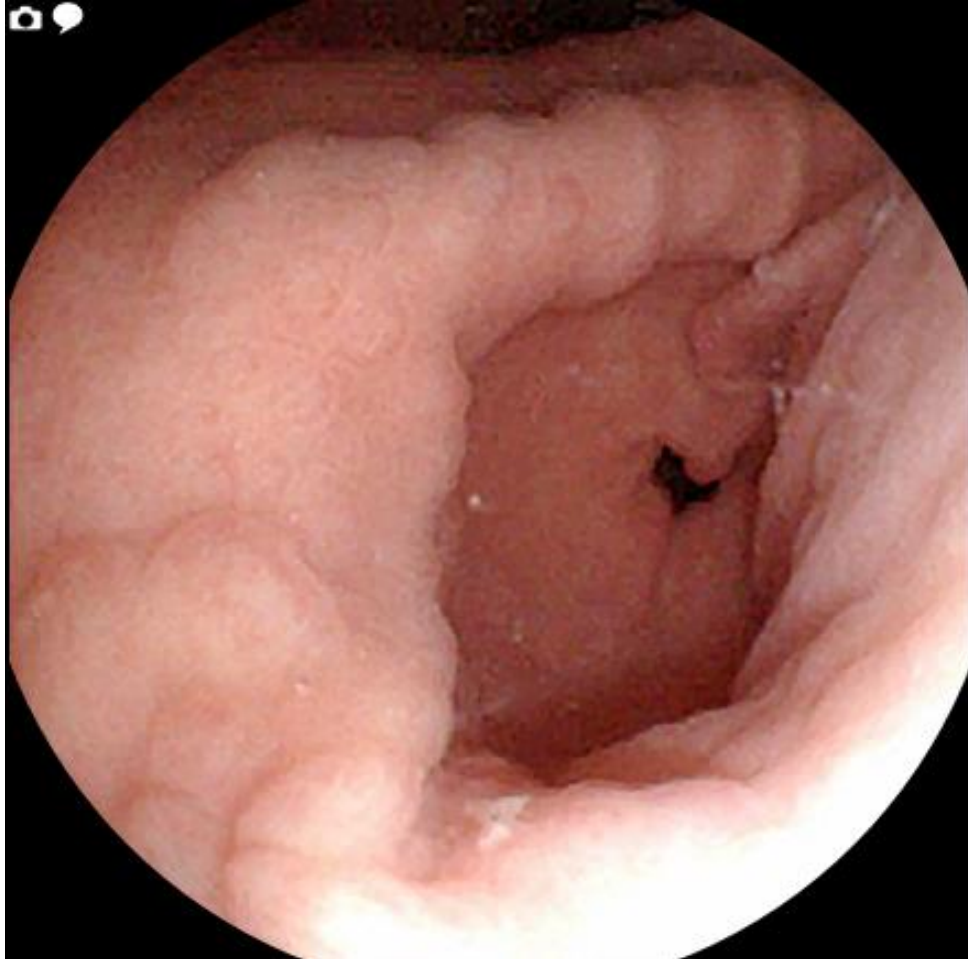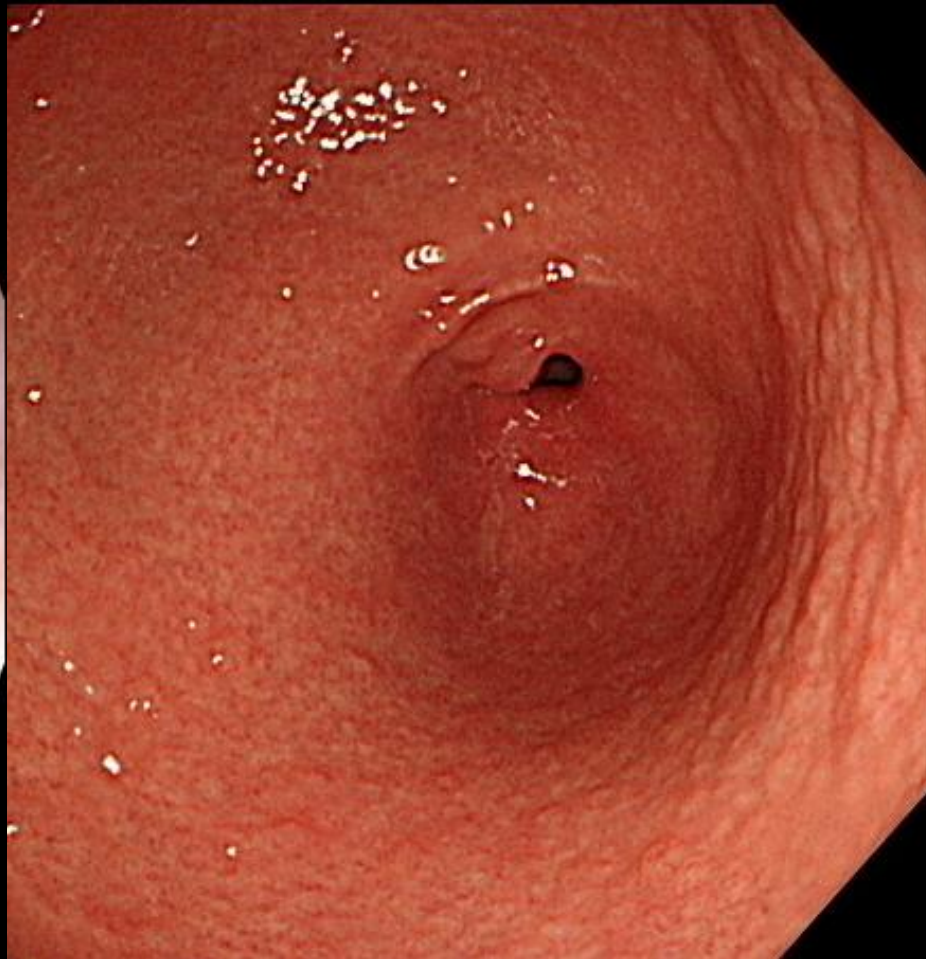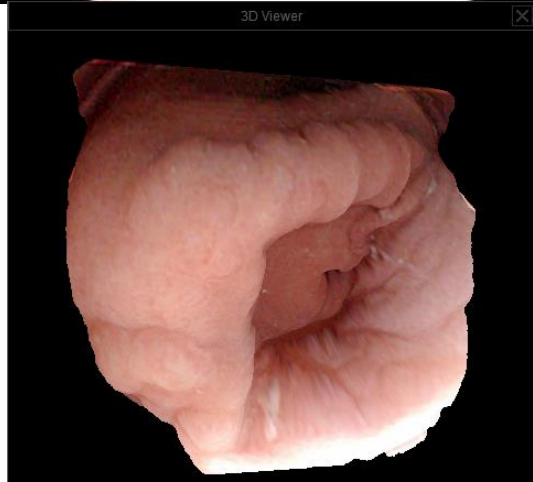

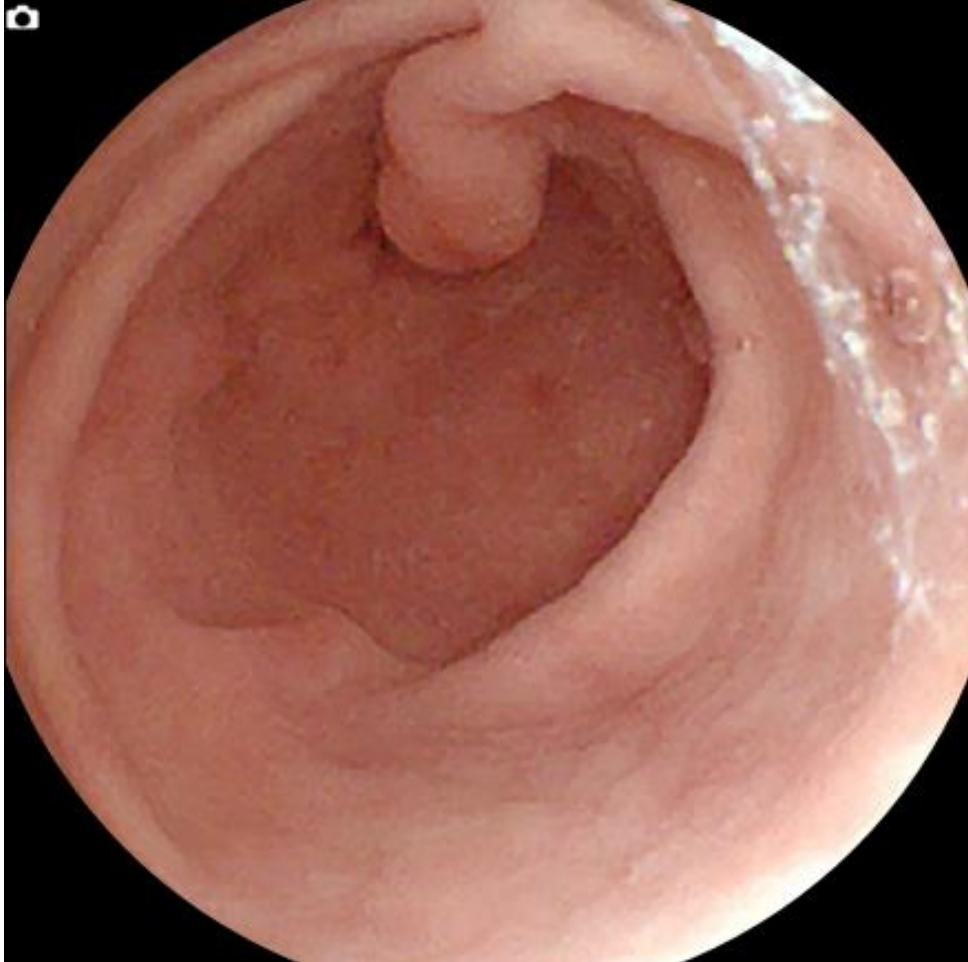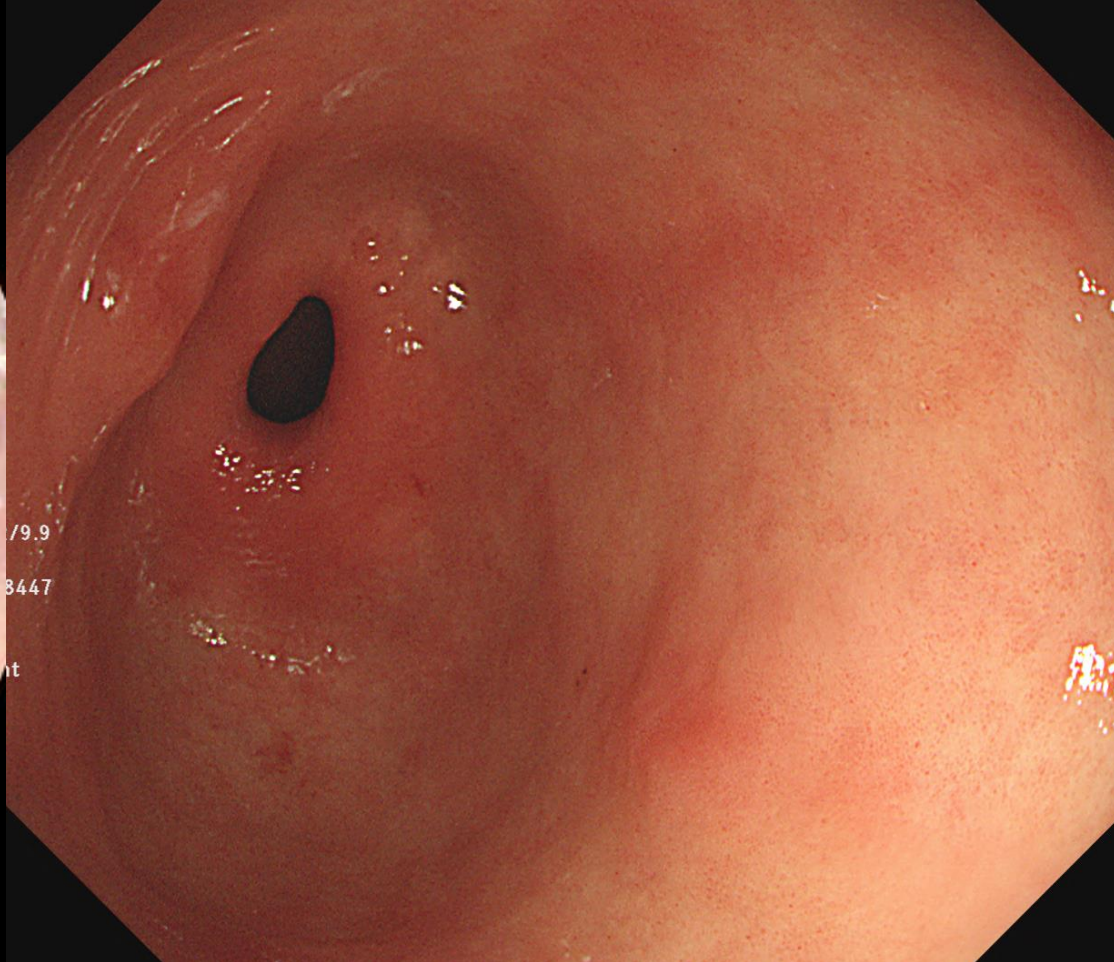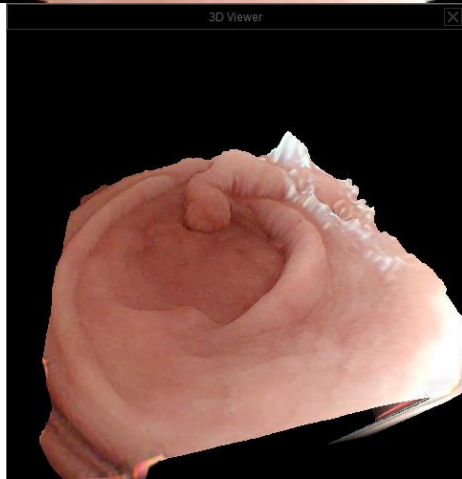

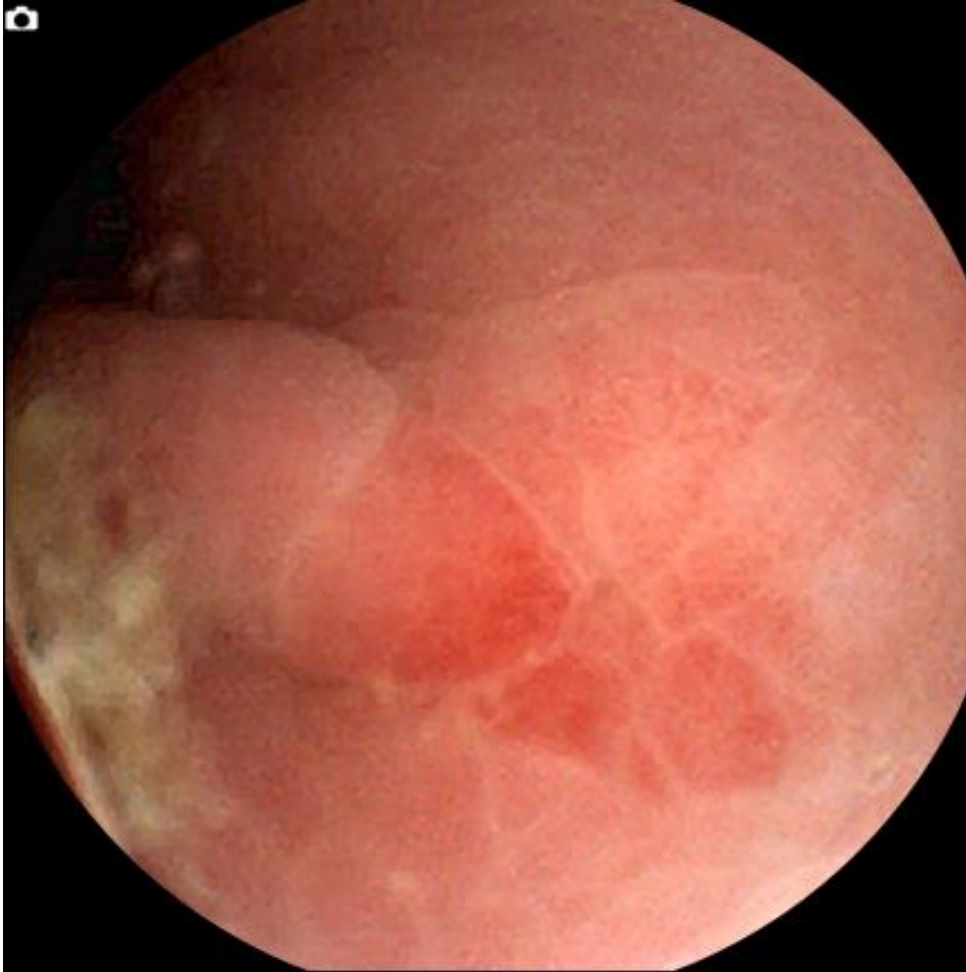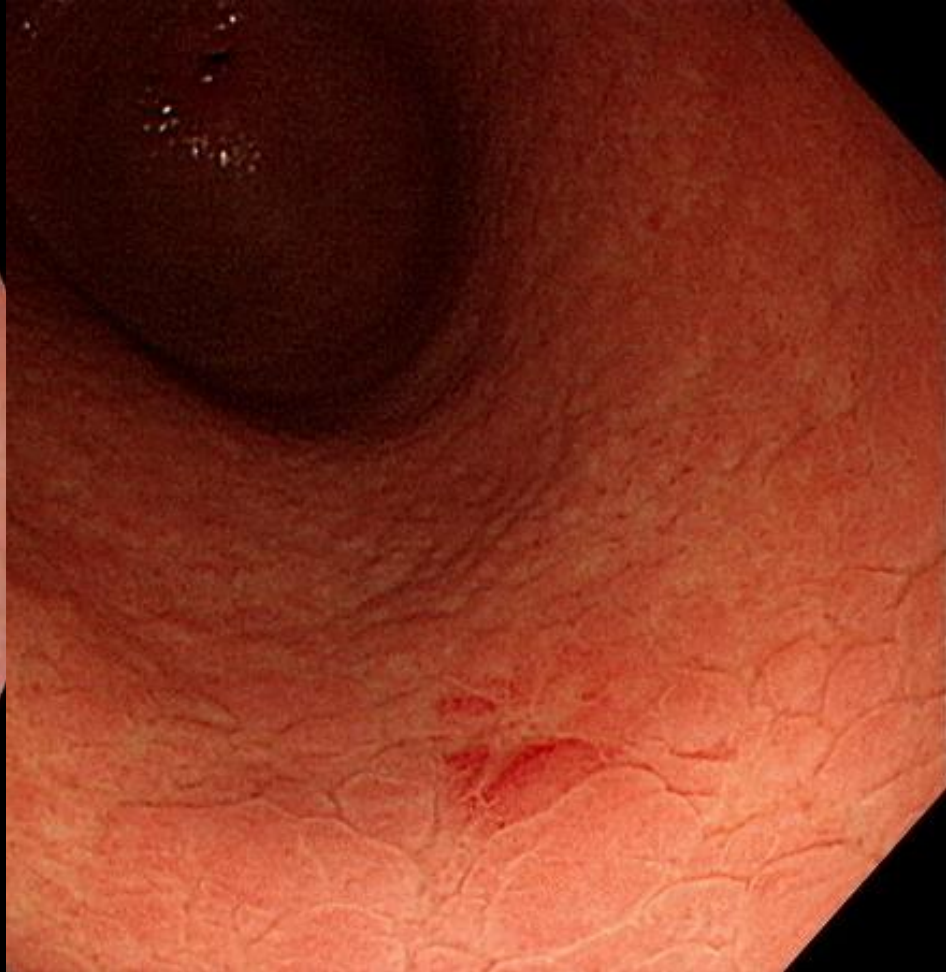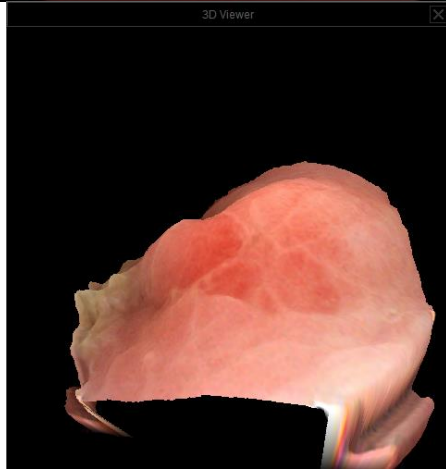

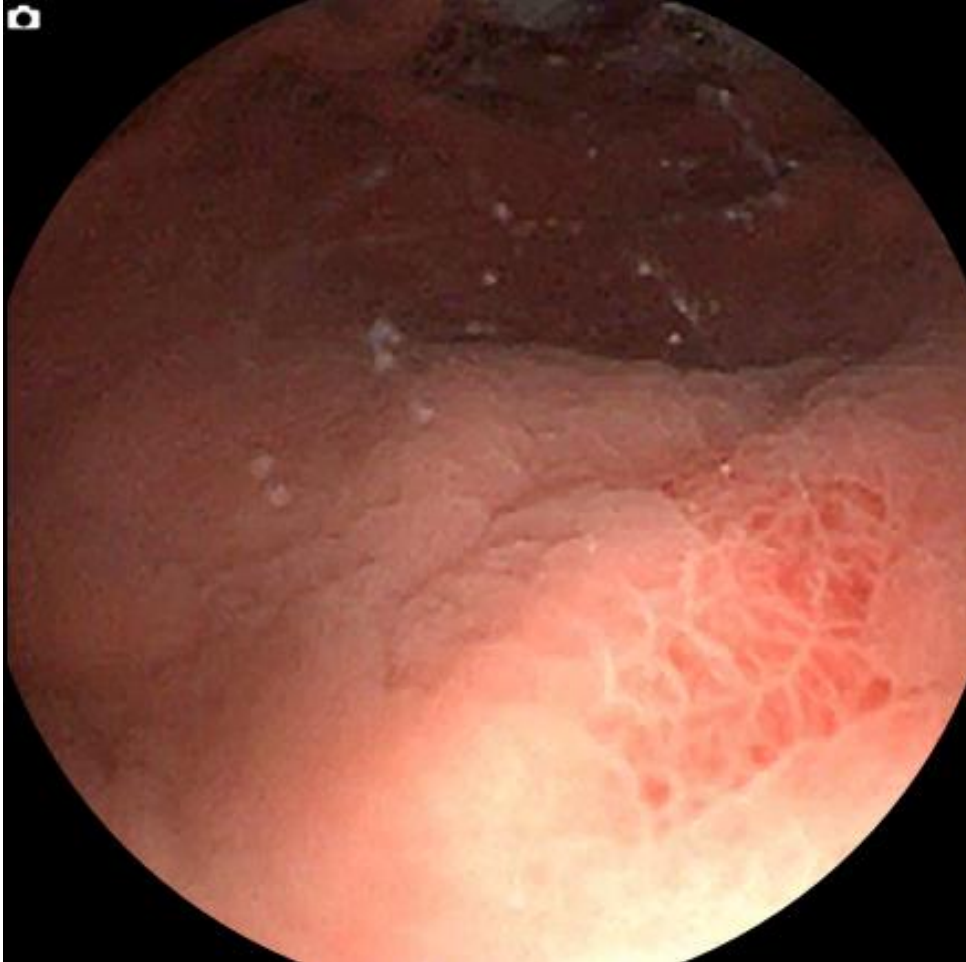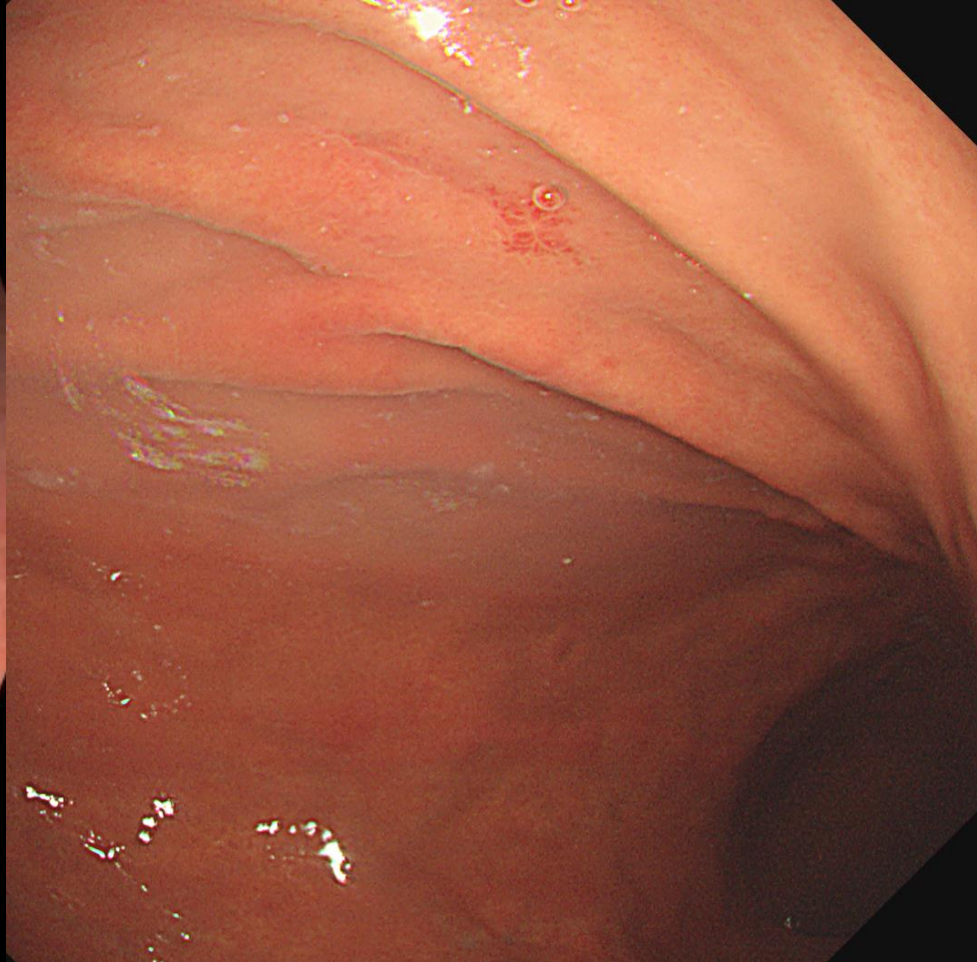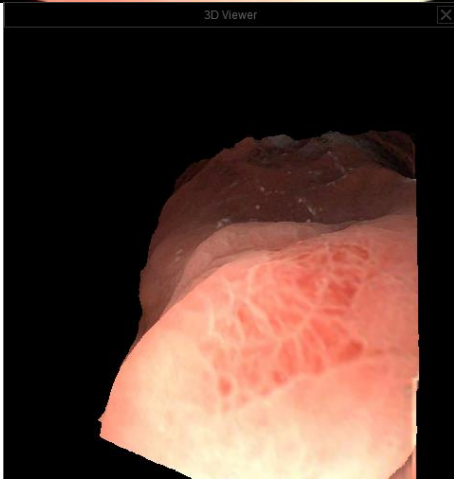

Supplement: S1 Dataset — (ZIP) [file pone.0295774.s006.zip › Lesion - 3D MACE and Upper endoscopy.pdf]

# Upper GI structure

Images of MACE (R001~R055)

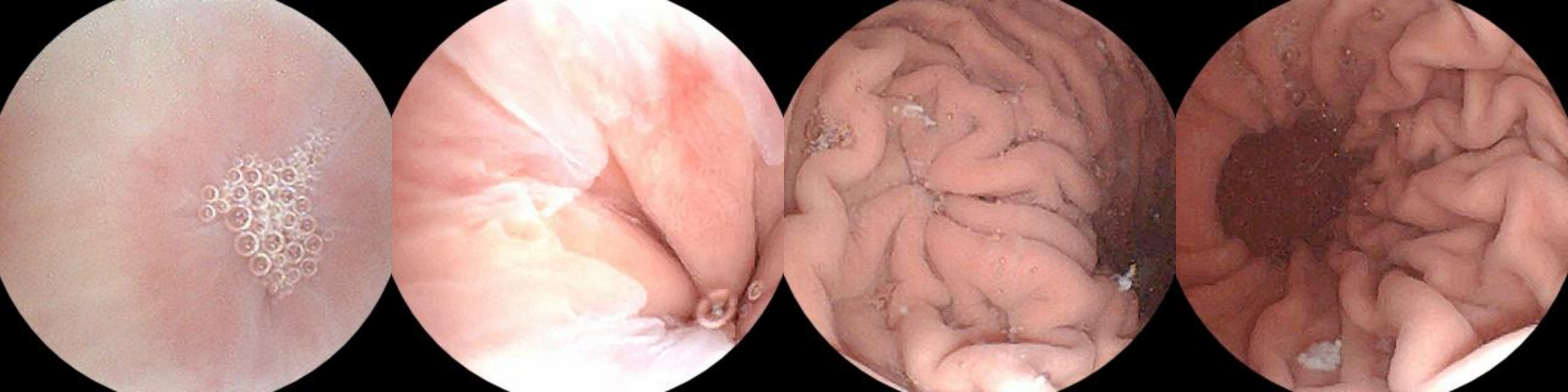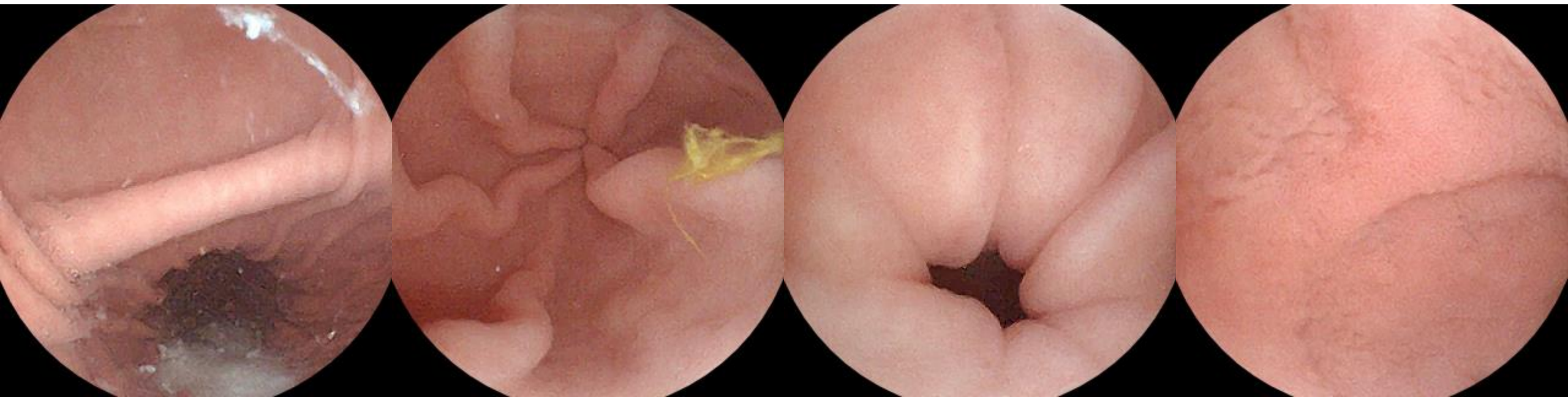

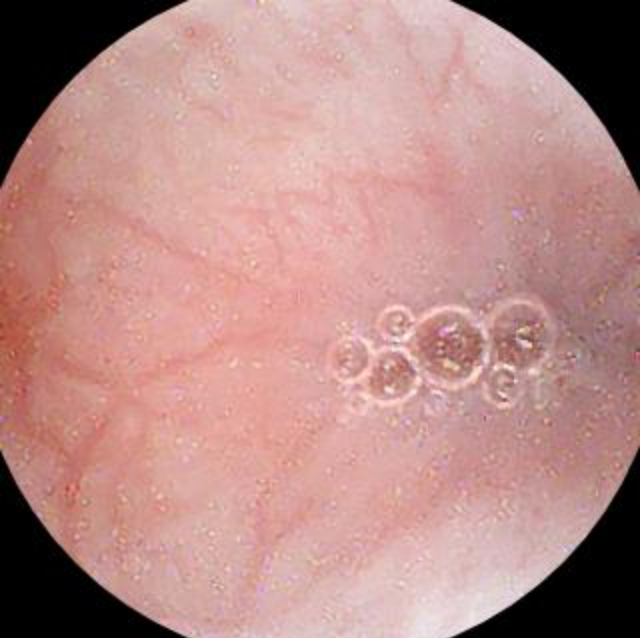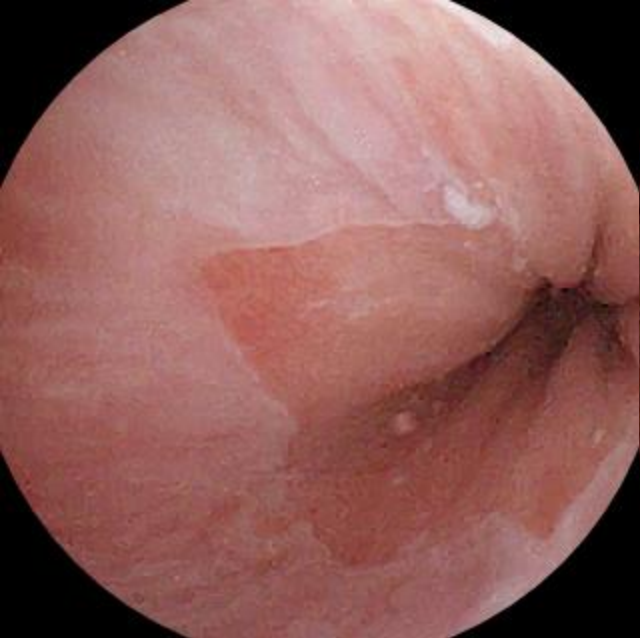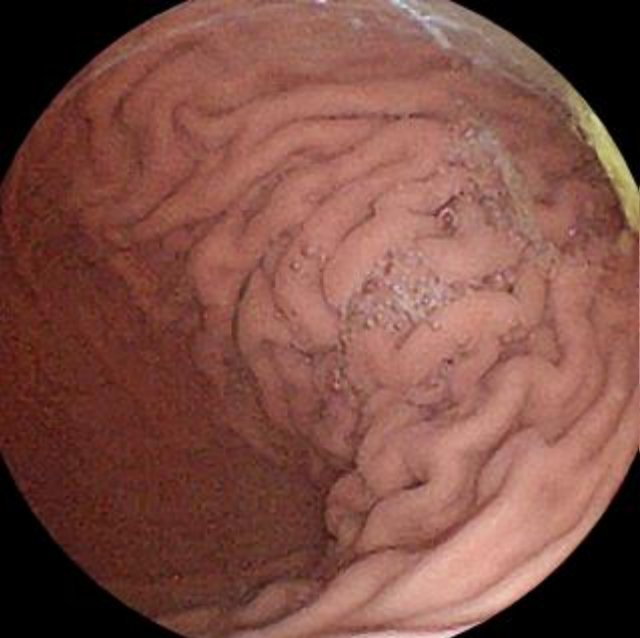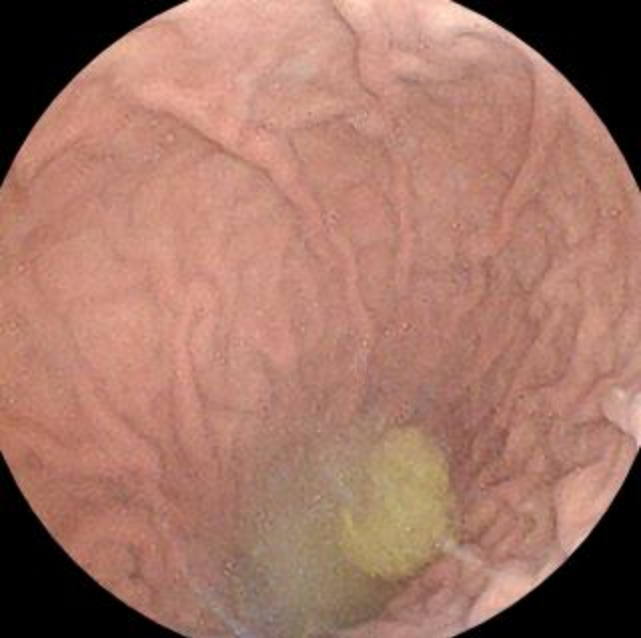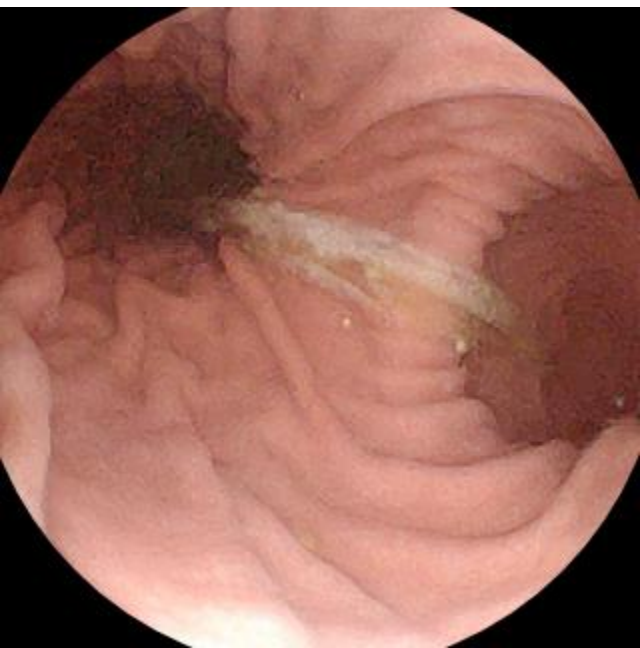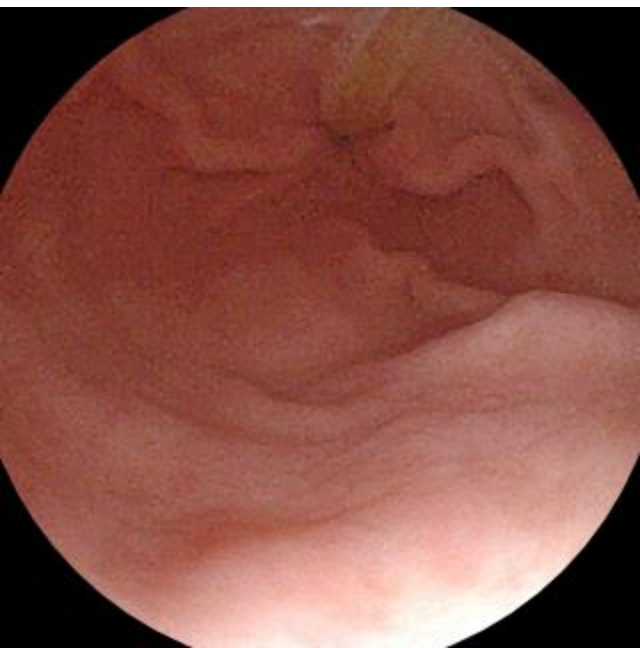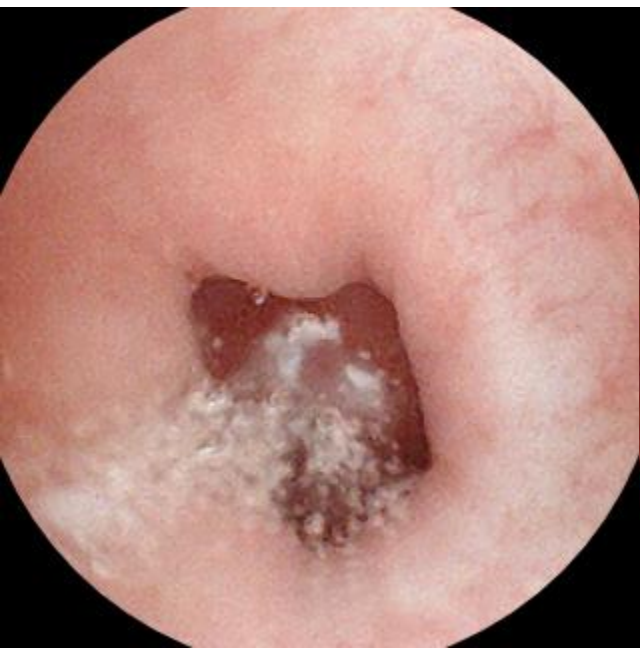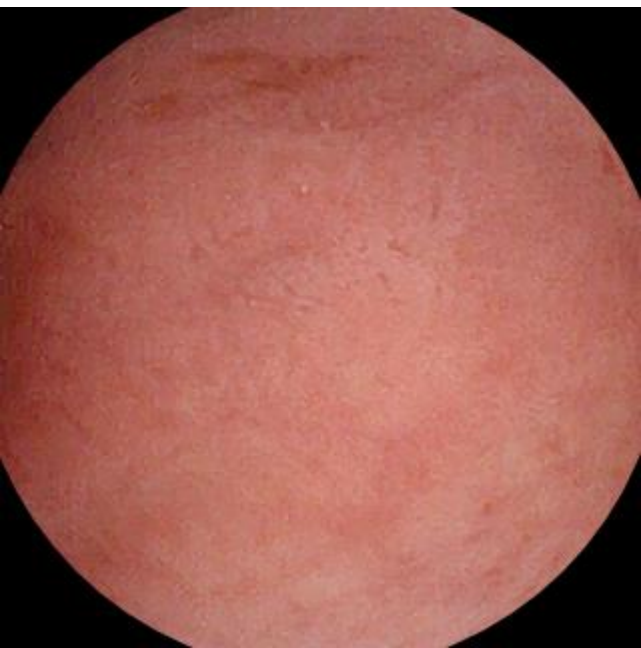

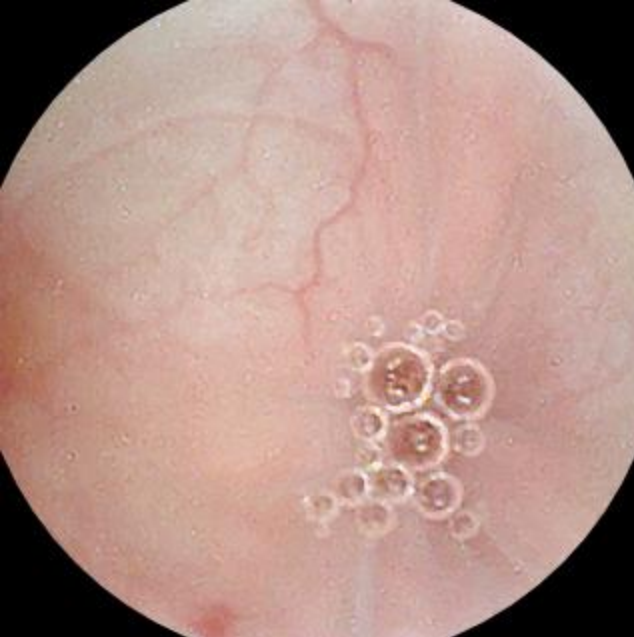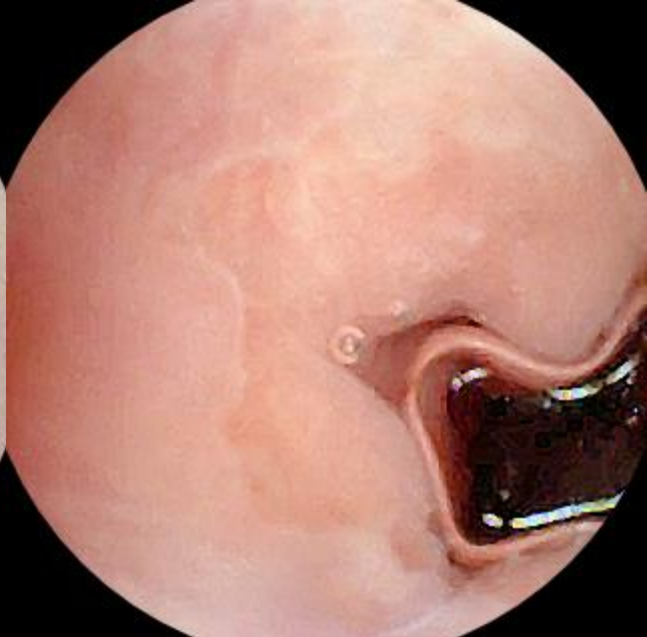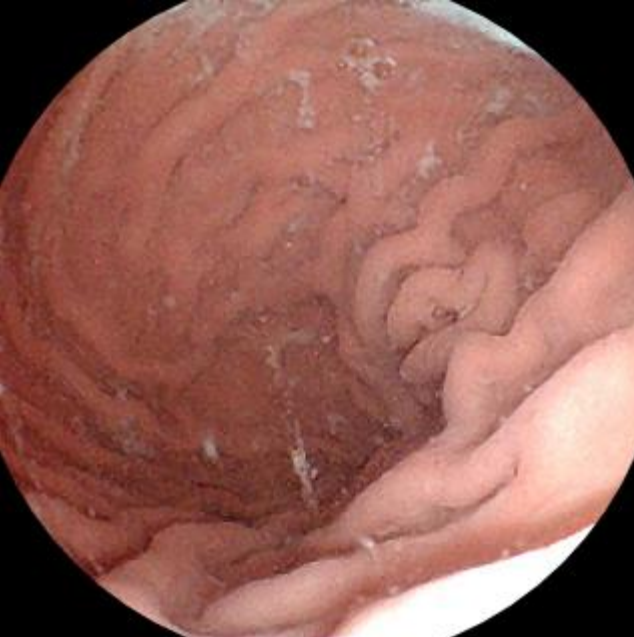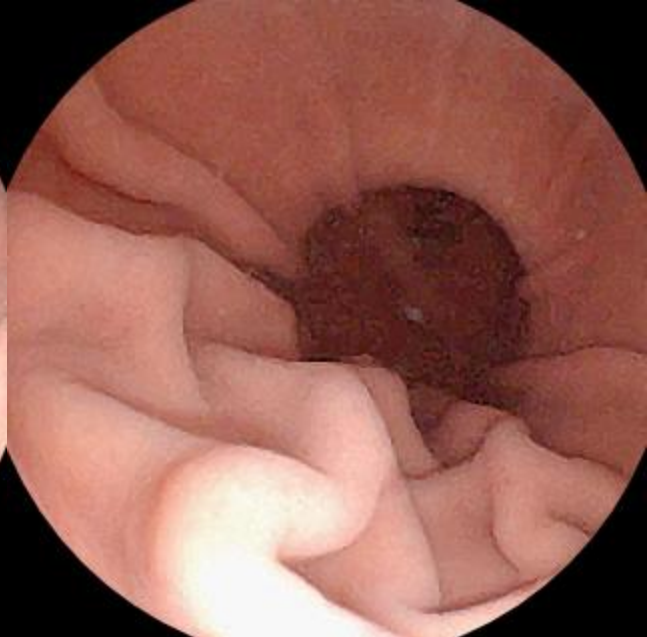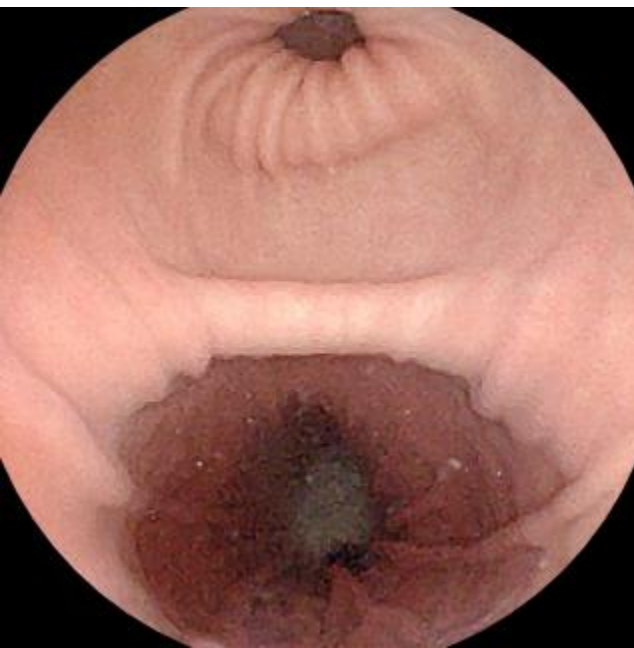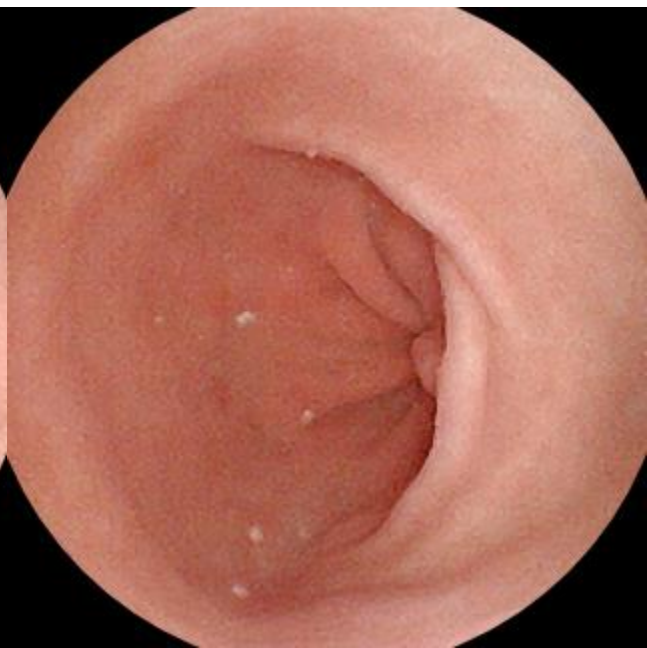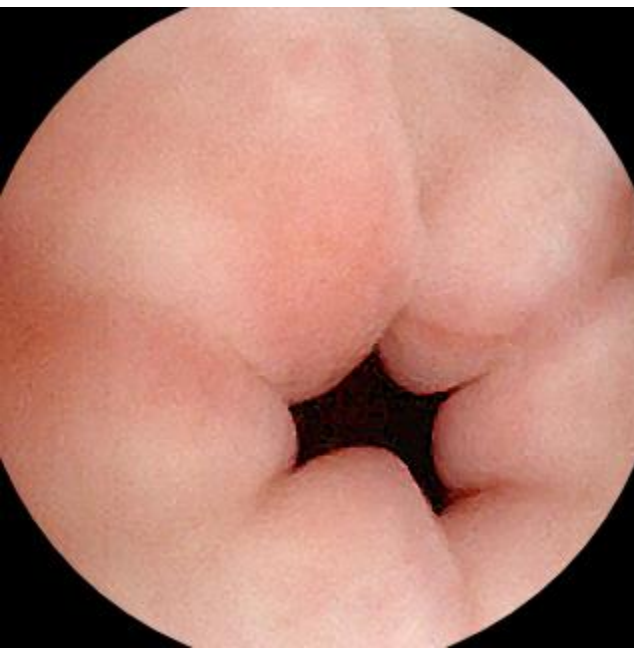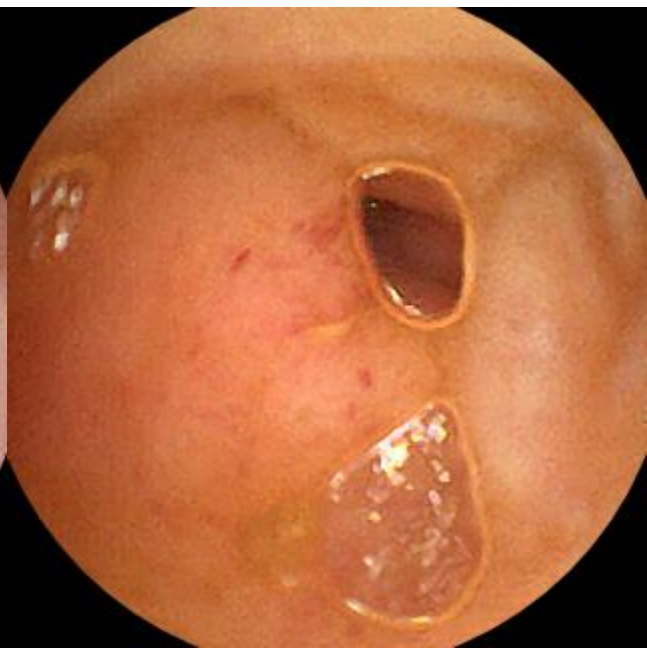

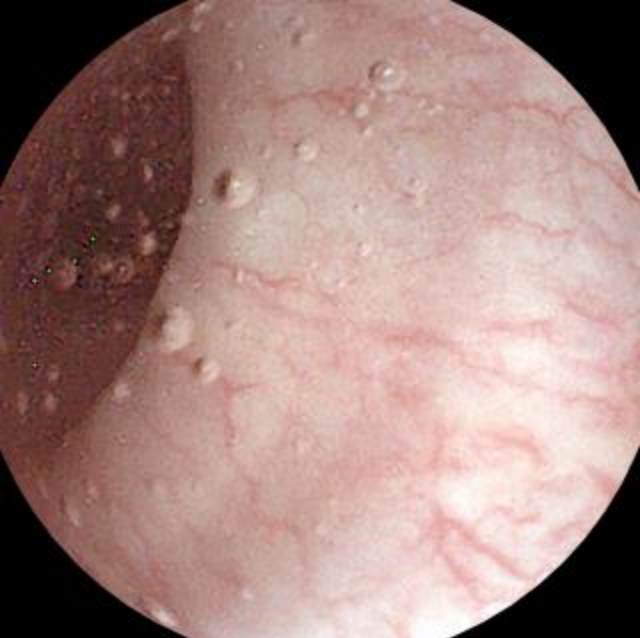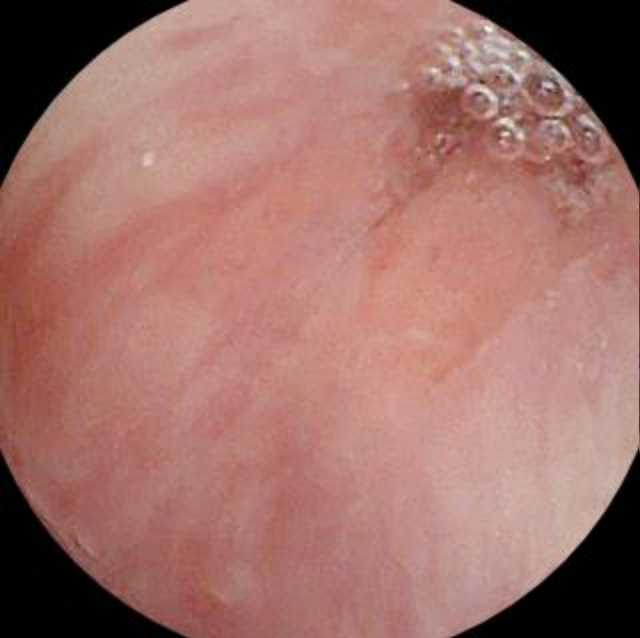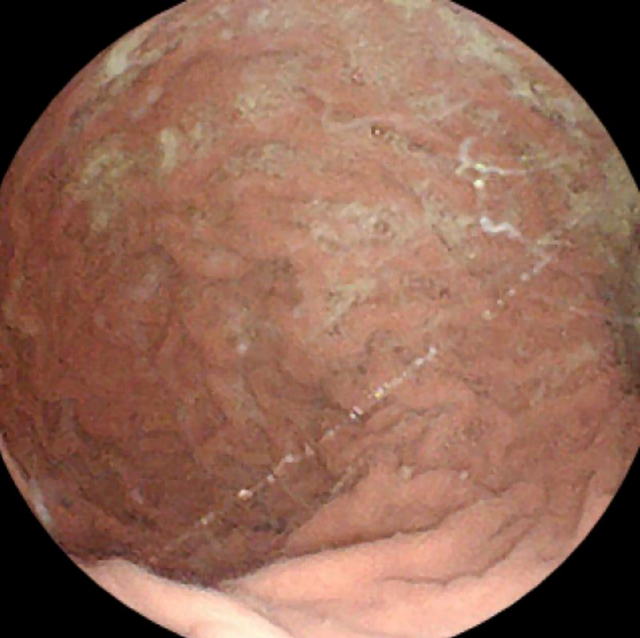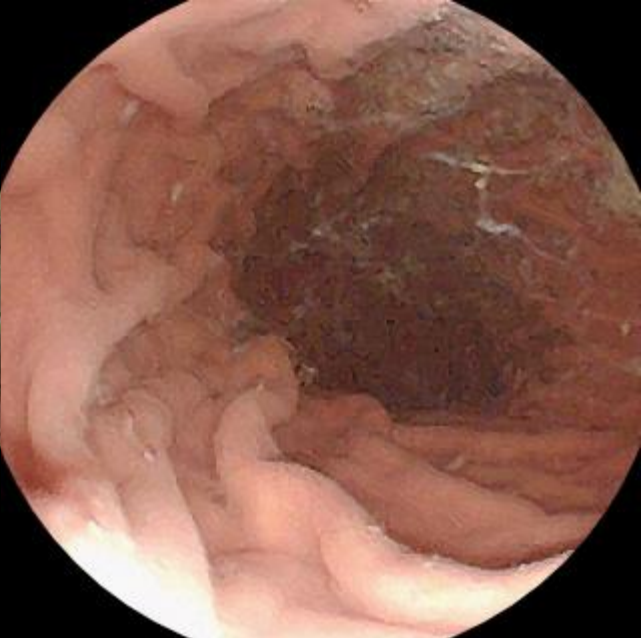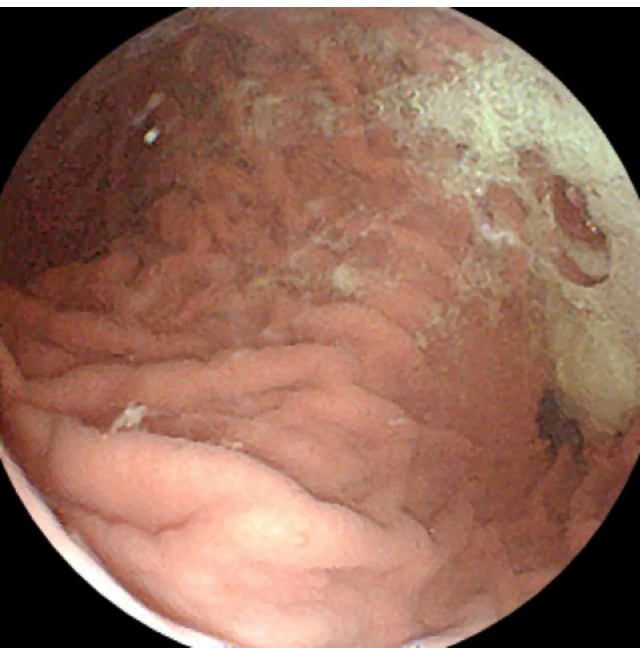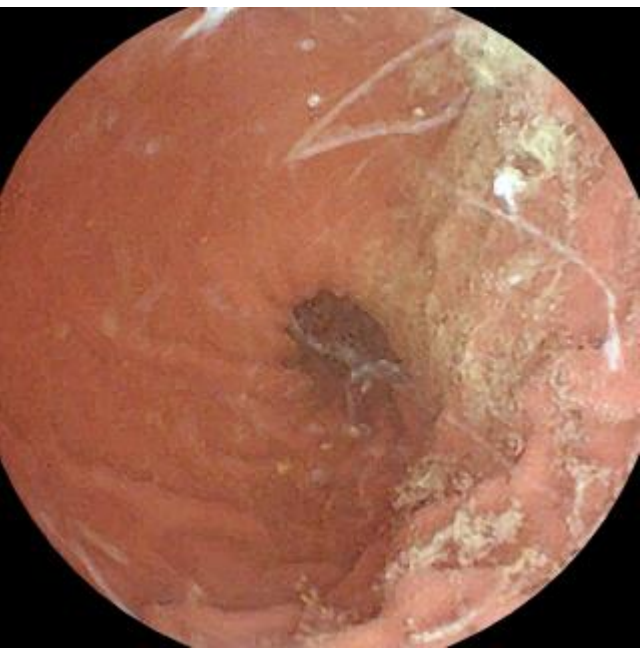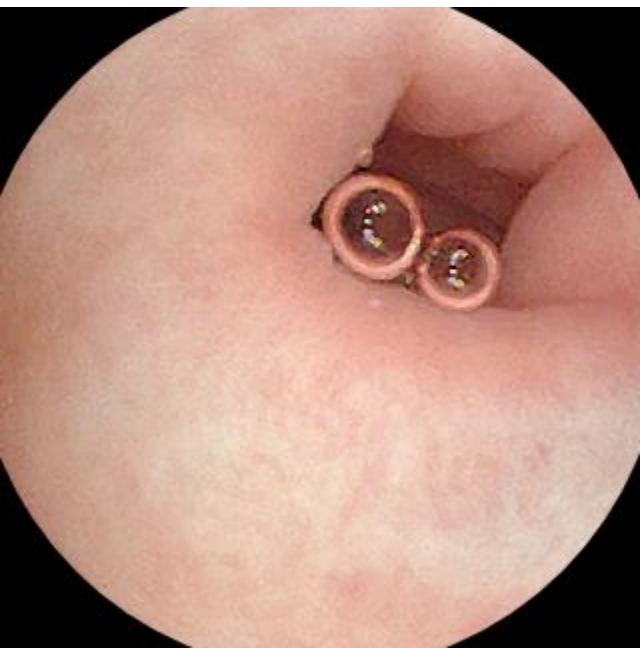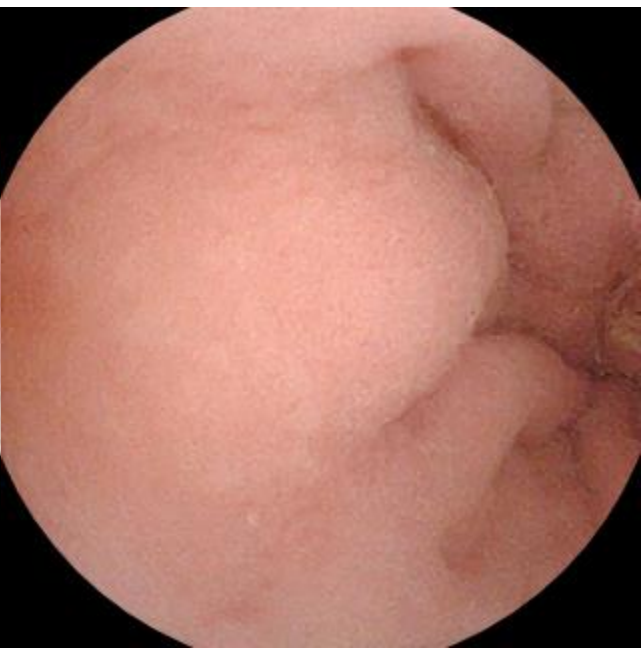

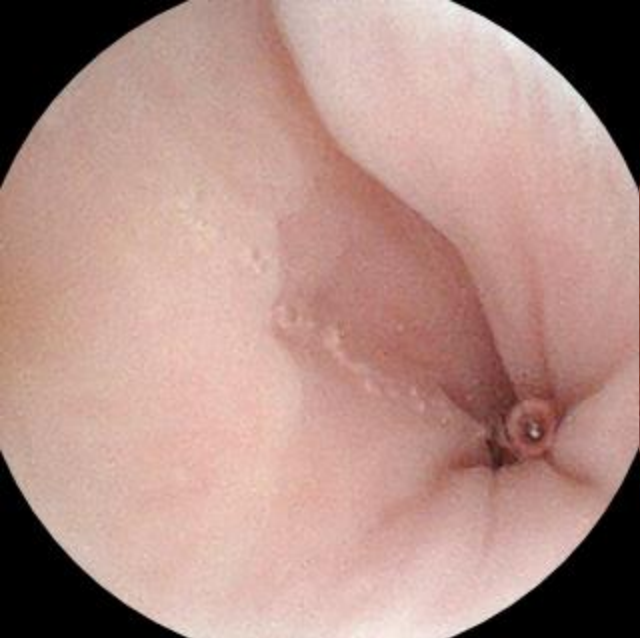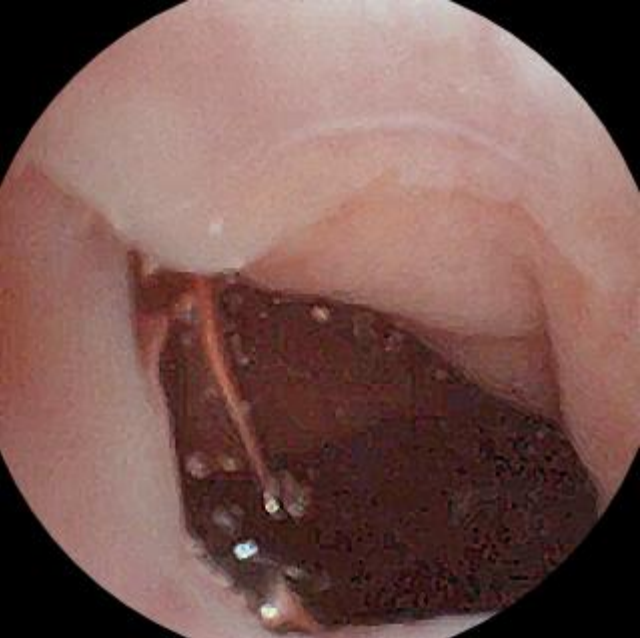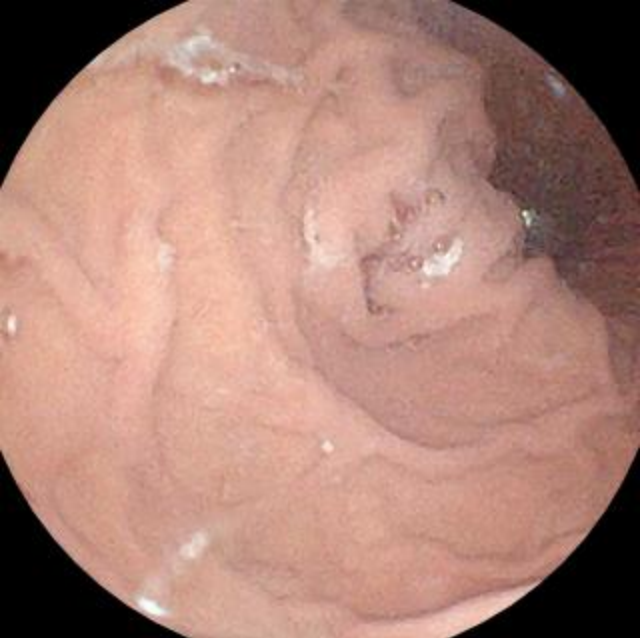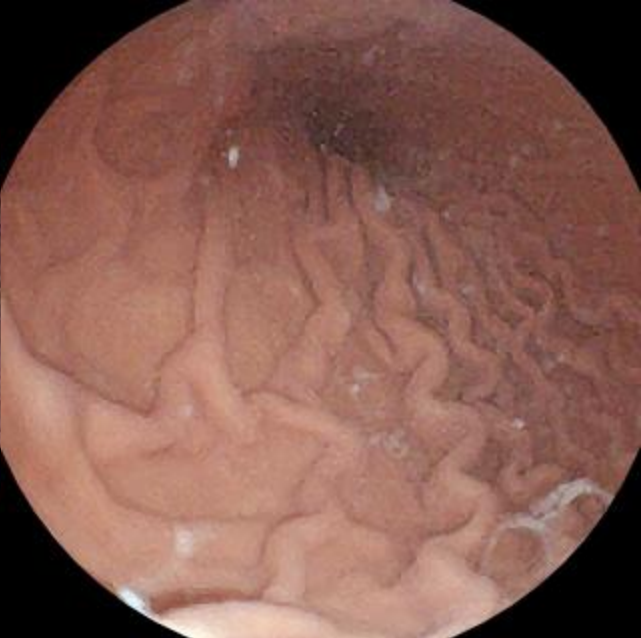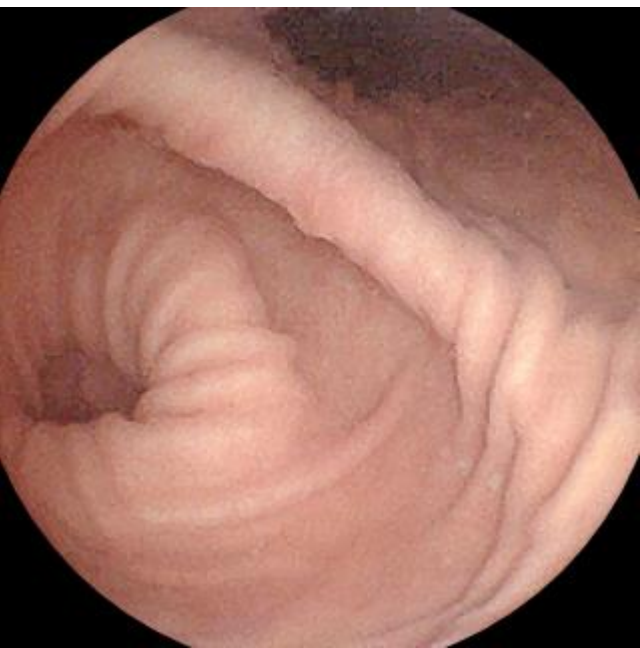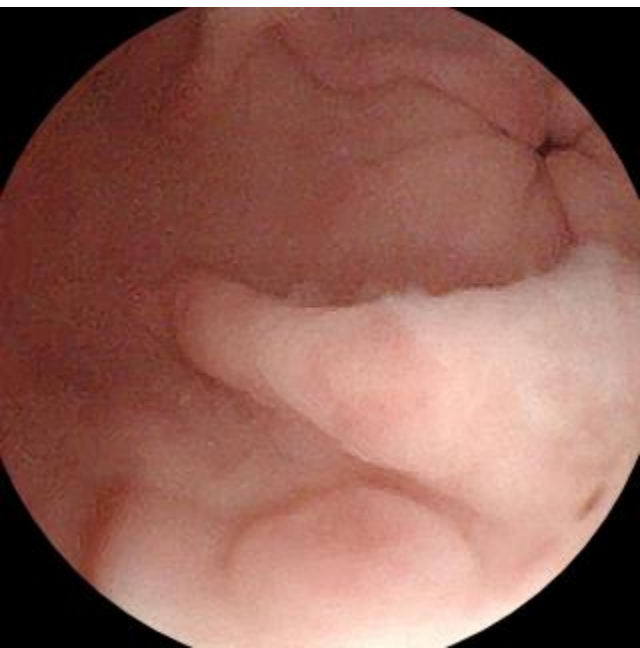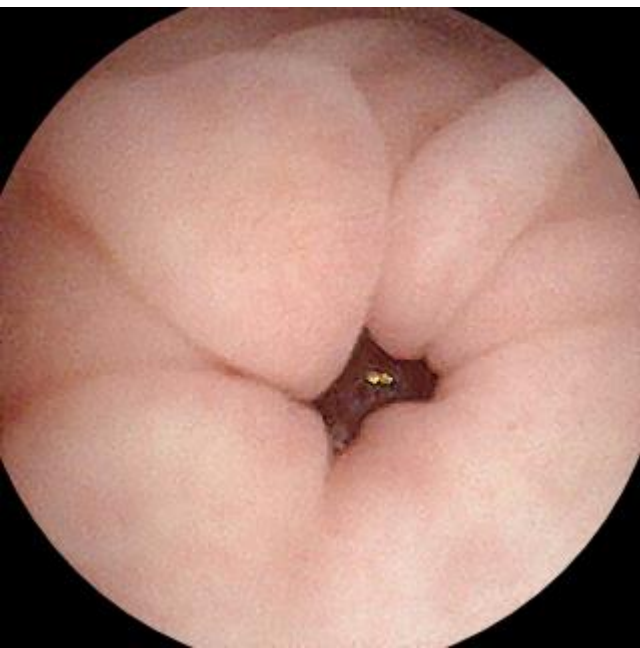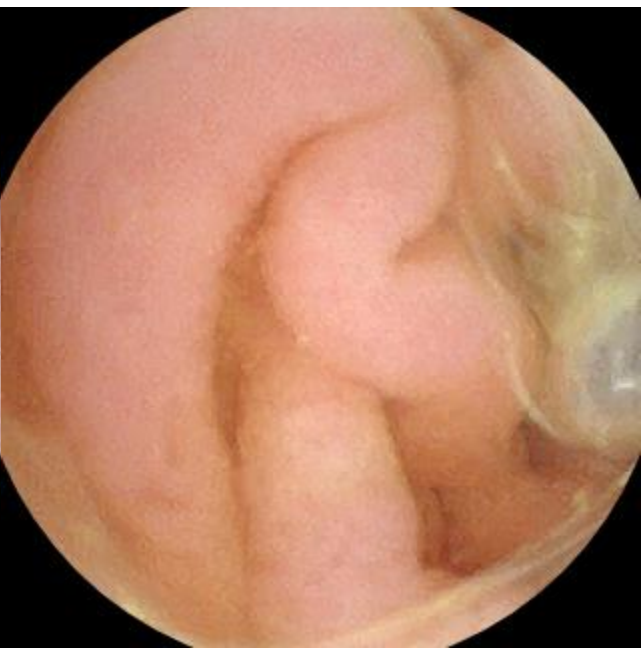

Supplement: S1 Dataset — (ZIP) [file pone.0295774.s006.zip › Upper GI structure - 3D MACE.pdf]
