# Supplementary material for: Efficacy and safety of three-dimensional magnetically assisted capsule endoscopy for upper gastrointestinal and small bowel examination
Source: PLoS One. 2024 May 7;19(5):e0295774. doi: 10.1371/journal.pone.0295774 (PMC11075891; doi:10.1371/journal.pone.0295774)
Supplement: S1 Table — (DOCX) [file pone.0295774.s004.docx]

**S3 Table. Baseline characteristics of study populations**

| Variables | Patients (n = 55) |
| --- | --- |
| Mean age, years (SD^a^) | 42.04 (± 10.36) |
| Female / Male | 28 (50.9%) / 27 (49.1%) |
| Body measurements |  |
| heights, cm (SD) | 168.74 (± 8.67) |
| weights, kg (SD) | 68.72 (± 13.08) |
| body mass index, kg/m2 (SD) |  |
| < 25 | 39 (70.9%) |
| ≥ 25 | 16 (29.1%) |
| Systolic / Diastolic blood pressure | 123.82 / 79.58 (± 13.08) |
| Heart rate (per min) | 81.56 (± 12.88) |
| Past medical history |  |
| hypertension | 4 (7.3%) |
| dyslipidemia | 3 (5.5%) |
| diabetes | 2 (3.6%) |
| hypothyroidism | 2 (3.6%) |
| cerebral infarction  asthma | 1 (1.8%)  1 (1.8%) |

^a^SD; standard deviation
