# Supplementary material for: Efficacy and safety of three-dimensional magnetically assisted capsule endoscopy for upper gastrointestinal and small bowel examination
Source: PLoS One. 2024 May 7;19(5):e0295774. doi: 10.1371/journal.pone.0295774 (PMC11075891; doi:10.1371/journal.pone.0295774)
Supplement: S2 Table — (DOCX) [file pone.0295774.s005.docx]

**S5 Table. Patient satisfaction during examination.**

| Discomfort | 3D MACE (n = 55) | Remark |
| --- | --- | --- |
| During examination |  |  |
| no | 53 (96.4%) |  |
| mild | 2 (3.6%) |  |
| moderate | 0 (0.0%) |  |
| severe | 0 (0.0%) |  |
| Other than examination^a^ |  |  |
| taking purgative | 34 (61.8%) |  |
| taking 3D MACE and water | 5 (9.1%) |  |
| returning the data sensor belt | 2 (3.6%) |  |
| Mean satisfaction score^b^ | 9.55±0.79 | 7.75±2.3  in upper endoscopy  (p <0.0001) |

^a^Duplicate voting possible

^b^Scales from 1 (very dissatisfied) to 10 (very satisfied)
