# Supplementary material for: Efficacy and safety of three-dimensional magnetically assisted capsule endoscopy for upper gastrointestinal and small bowel examination
Source: PLoS One. 2024 May 7;19(5):e0295774. doi: 10.1371/journal.pone.0295774 (PMC11075891; doi:10.1371/journal.pone.0295774)
Supplement: Attachment — Submitted filename: Response to reviewers.docx [file pone.0295774.s008.docx]

**Response to reviewers**

Thank you very much for giving the opportunity of revision. The authors appreciate the reviewer’s critical and thoughtful comments. We did our best to write the reply for your comments. We also used the "Track Changes" feature to make revision to the manuscript and make them easily visible to editors and reviewers.

**[Reviewer 1]**

1. Clarify more in the results besides detection, is it possible for the doctors to evaluate the characteristics of the lesions using the standardized classification? For the inflammatory lesions, can 3D MACE detect both the lesions with focal and spreading areas? If possible adding more characteristics of the lesions and endoscopists' evaluations will make the data more interesting.

: This is an important point. Of course, 3D MACE operator and endoscopist each classified the characteristics of the inflamed lesion such as erosive, hyperemic, and edematous. However, subgroup analysis was not performed because there were differences between 3D MACE operator and endoscopist in distinguishing whether an inflamed lesion was hyperemic or erosive. Additionally, this distinction was thought to be minor and insignificant, and was not relevant to the outcome of this study.

: The extent of the inflamed lesion could be confirmed similarly for both 3D MACE and upper endoscopy. This information added to the “Result; Table 2” and “Discussion; Limitations” section.

2. For the cases with mucosal injury and minor bleeding, in fact what is the origin of the bleeding? EGD is safe so it is a bit confusing why patients have post-procedure bleeding?

: Most procedure related bleeding involved belching in this study. The bleeding was spontaneously stopped without any additional procedure. We added this content to the “Result; Safety and patient satisfaction” section.

**[Reviewer 2]**

1. Financial disclosure - Intromedic is mentioned as clinical sponsor in the study protocol which is not stated in the financial disclosure. Please clarify Intromedic's role in the study

: Intromedic company only developed 3D MACE for a research project and we did the research independently. The company had no research or financial involvement in this study. Additionally, the authors have no financial or personal relationship with Intromedic. We added this content in the “Acknowledgments” section.

2. Method - Further description on manipulation of the MACE after ingestion should be provided.

: We added a description of the manipulation of 3D MACE in “Method; Study protocol; 3D MACE for upper GI and small bowel examination” section

→ “When the 3D MACE stopped at the esophagus, the controller was moved up and down to observe the esophagus.” “Once the 3D MACE landed on the stomach, the cardia and fundus were observed by rotating and tilting the controller in the left lateral position. Then, the patient was placed upright, and the controller was placed in the left upper quadrant and left flank area to observe the fundus and body. After that, the operator moved the controller to right upper quadrant and right flank area to observe the antrum and pylorus. If necessary, the patient was changed to the right lateral position and the antrum was observed.”

3. Method - Please define unsuccessful examination of anatomical structure or suspected lesions eg. length of time or number of attempts in assessment of a structure or lesion of concern

: This is a good point. Mucosal visualization for each structure was also assessed. Failure to structure confirmation was defined as a case where the structure was not visible during examination or was not clearly photographed. There were no restrictions on length of time or number of attempts. We added this content in the “Method; Outcomes and Safety” section.

4. Result and discussion - Photos taken by MACE vs. 3D MACE vs. conventional endoscopy on selected case should be provided for better appreciation by the readers

: MACE and 3D MACE are completely different models. And since this study was approved for clinical research only using 3D MACE, there is no data regarding MACE. Therefore, MACE and 3D MACE could not be compared in this study. Instead, we will post here images of MACE published in other articles, for comparison with 3D MACE.

5. Result and discussion - For figure 5 regarding lesions detected, please comment on whether further assessment of lesions ie. image magnification, image enhancement is possible. Also, how to decide on need for conventional upper endoscopy with biopsy if the 3D-MACE is to be used independently.

: Image brightness and definition was enhanced using a reading viewer. We added this content in the “Figure 5” section. Gastric examination using a magnetic capsule is still in the challenging stage. Therefore, it is considered premature to separate 3D MACE from upper endoscopy. The need for biopsy in 3D MACE is also considered to be the same as the indications for upper endoscopy such as gastric ulcer and suspected gastric cancer. We added this content in the “Discussion; Limitation” section.

6. Result and discussion - Image on small bowel examination including detected pathology should be provided

: Images of small bowel ulcers were included in S4 Fig.

7. Result and discussion - Regarding the 3 inflamed lesions and 1 polyps detected by 3D MACE but not upper endoscopy, how are the cases managed? Are they considered as false positive or follow up assessment is arranged to confirm the lesion?

: Thank you for your thoughtful comment. In the case of missed lesions, the authors reviewed the 3D MACE images and concluded that the lesions were insignificant. The endoscopist explained that the patients needed periodic follow-up endoscopy. We added this information in the “Result” section.

8. Result and discussion - Additional limitation of the study is no gastric cancer cases being recruited, therefore efficacy in detecting potential malignant lesion cannot be inferred

: I agree with your comment. We added to the “Discussion” section the limitation that we could not confirm the efficacy of gastric cancer detection because we did not have gastric cancer patients.
