# Supplementary material for: Efficacy and safety of three-dimensional magnetically assisted capsule endoscopy for upper gastrointestinal and small bowel examination
Source: PLoS One. 2024 May 7;19(5):e0295774. doi: 10.1371/journal.pone.0295774 (PMC11075891; doi:10.1371/journal.pone.0295774)
Supplement: Attachment — Submitted filename: Response to reviewer - 2nd revision.docx [file pone.0295774.s009.docx]

**Response to reviewer**

Thank you very much for giving the opportunity of revision. The authors appreciate the reviewer’s critical and thoughtful comments. We did our best to write the reply for your comments. We also used the "Track Changes" feature to make revision to the manuscript and make them easily visible to editors and reviewer.

**[Reviewer 3]**

On the overall, the authors were able to address the previous round of comments. Some inconsistencies are still present in the statistical section:

1. The power/sample size stab does not mention the statistical test that was used.

: Thank you for the helpful suggestion. We revised the sentence as follows in relation to mention of power/sample size stability: ‘To ensure the power and stability of the sample size in our study, we conducted a Power Analysis, setting the level of significance (α) at 0.05 and the power of the test (β) at 80%. This involved considering a standard deviation of 4.0 based on the successful visualization of gastric structures from the previous study [5], and an effect size of 0.8 in the t-test for the Power Analysis.

2. It is not clear what the authors meant by "Kapp" in "Kapp, sensitivity, and specificity were used to assess..."

: Kapp is a misspelling of kappa (k), and I apologize for the mistake. We revised the sentence as follows: ‘Kappa (κ), sensitivity, and specificity were used to evaluate the agreement and assess each part of upper GI anatomy and gastric lesions between two groups.
